# Associations of a current Australian model of dietetic care for women diagnosed with gestational diabetes and maternal and neonatal health outcomes

**DOI:** 10.1186/s12913-023-09924-4

**Published:** 2023-09-08

**Authors:** Gina Absalom, Julia Zinga, Claire Margerison, Gavin Abbott, Sharleen O’Reilly, Paige van der Pligt

**Affiliations:** 1 School of Exercise and Nutrition Sciences, Deakin University, Geelong, VIC 3220 Australia; 2 Department of Nutrition & Dietetics, Royal Women’s Hospital, Parkville, VIC Australia; 3 Institute for Physical Activity and Nutrition (IPAN), School of Exercise and Nutrition Sciences, Deakin University, Geelong, Australia; 4 School of Agriculture and Food Science, University College Dublin, Belfield, Dublin 4 Ireland

**Keywords:** Gestational diabetes mellitus, Pregnancy, Neonate, Nutrition, Health service, Maternal, Dietitian

## Abstract

**Background:**

Gestational diabetes mellitus (GDM) is a significant public health burden in Australia. Subsequent strain on healthcare systems is widespread and current models of care may not be adequate to provide optimal healthcare delivery. This study aimed to assess a current model of dietetic care with maternal and neonatal outcomes.

**Methods:**

Hospital medical record data from The Women’s Hospital, Melbourne, for women with GDM (n = 1,185) (July 2105-May 2017) was retrospectively analysed. Adjusted linear and logistic regression were used to analyse associations between the number of dietitian consultations and maternal and neonatal health outcomes.

**Results:**

Half of all women (50%) received two consultations with a dietitian. 19% of women received three or more consultations and of these women, almost twice as many were managed by medical nutrition therapy (MNT) and pharmacotherapy (66%) compared with MNT alone (34%). Higher odds of any maternal complication among women receiving 3 + consultations compared to those receiving zero (OR = 2.33 [95% CI: 1.23, 4.41], p = 0.009), one (OR = 1.80 [95% CI: 1.09, 2.98], p = 0.02), or two (OR = 1.65 [95% CI: 1.04, 2.60], p = 0.03) consultations were observed. Lower odds of infant admission to the Neonatal Intensive Care Unit (NICU) were observed among women receiving one (OR = 0.38 [95% CI: 0.18, 0.78], p = 0.008), two (OR = 0.37 [95% CI: 15 0.19, 0.71], p = 0.003), or three + consultations (OR = 0.43 [95% CI: 0.21, 0.88], p = 0.02), compared to no consultations.

**Conclusion:**

The optimal schedule of dietitian consultations for women with GDM in Australia remains largely unclear. Alternate delivery of education for women with GDM such as telehealth and utilisation of digital platforms may assist relieving pressures on the healthcare system and ensure optimal care for women during pregnancy.

## Background

Gestational diabetes mellitus (GDM) affects 1 in 5 pregnancies globally [1, 2] with prevalence found to be as high as 28% in some population groups [3]. In 2017, 15% of pregnancies in Australia were affected by GDM [1] with prevalence rates having quadrupled over the last decade [4]. Consequently, healthcare service pressures have increased dramatically [5] and antenatal clinicians are faced with increased challenges in service provision and in meeting the needs of diverse population groups diagnosed with GDM in Australia [4].

A diagnosis of GDM during pregnancy increases risk for multiple adverse maternal and neonatal health outcomes including pre-eclampsia, [6, 7] macrosomia, pre-term birth and caesarean delivery [8, 9]. In addition, the cost burden on healthcare systems resulting from increased resource requirements is substantial. In Australia, women diagnosed with GDM have significantly higher in-hospital service usage compared to women without GDM [10]. This includes increased emergency c-section delivery, longer length of hospital stay, higher neonatal complications requiring additional intervention and greater health service utilisation, including specialist and allied health, input; all of which contribute to higher health costs associated with managing women with GDM compared to those without [11, 12].

Current models of GDM care have been considered “unsustainable” in Australian healthcare settings [4]. While not all women diagnosed with GDM require the same level or intensity of management, the delivery of GDM care is workforce intensive [5]. GDM care requires input from diabetes educators, endocrinologists, and dietitians [5, 13] to adequately support women to engage in glucose monitoring, physical activity, medical nutrition therapy (MNT) and pharmacotherapy, where hyperglycaemia persists [14]. Dietitian-delivered MNT is recommended as a first-line therapy for women diagnosed with GDM [13] and is considered the foundation for ongoing management [4]. Yet, the current model of MNT is time intensive and not sustainable for clinicians or pregnant women.

Currently no Australian clinical practice guidelines exist for the dietetic intervention that women should receive when diagnosed with GDM. U.S. evidence-based guidelines [15] include a recommended appointment schedule for early and frequent dietetic intervention, specifically recommending a minimum of three dietetic consultations be provided during pregnancy, with the first two visits occurring within the first two weeks of GDM diagnosis and additional consultations offered if necessary [15]. Adherence to these guidelines has been shown to reduce the need for insulin therapy, but no effect on neonatal outcomes such as infant birthweight [16]. Where staff resources are limited, questions have been raised regarding the feasibility and impact of a universal appointment schedule, this concern applies to most Australian dietetic services [17]. Better understanding the effectiveness of current models of GDM care is crucial to inform practical solutions which best target both preferable health outcomes for women alongside efficient and sustainable use of health service resources. This study aimed to assess a standard dietetic model of care for women with GDM at large, Australian maternity hospital and assess associations with maternal and neonatal health outcomes.

## Methods

### Study design and participants

This was a retrospective study of women (≥ 18 years of age) diagnosed with GDM (n = 1509) who gave birth between 1 and 2015 and 31 May 2017 at The Women’s Hospital, Melbourne. Ethics approval for this study was obtained by the Human Research Ethics Committee at The Women’s Hospital (17/08) and the Deakin University Human Research Ethics Committee (2017 − 190). Diagnosis of GDM was in accordance with the Australasian Diabetes in Pregnancy Society (ADIPS) (2014) criteria [18]. Dates of GDM diagnosis were obtained from pathology records (n = 729) and a manual audit of diabetes educators’ records (n = 780). ‘Early diagnosis (< 18 weeks gestation)’ or ‘routine diagnosis (≥ 18 weeks gestation)’ was documented. Women diagnosed ‘early’ with GDM were managed in the specialist diabetes clinic by an endocrinologist, obstetrician, dietitian and diabetes educator, as opposed to women diagnosed ≥ 18 weeks gestation, who remained in routine antenatal care by an obstetrician, dietitian and diabetes educator. In addition to consulting ‘early diagnosed’ women, the specialist diabetes clinic consulted women deemed to have ‘higher risk’ GDM. Higher risk was defined as: HbA1c ≥ 6.5%; or overt diabetes on glucose tolerance test: fasting blood glucose level ≥ 7.0mmol/L and/or 2-h blood glucose level ≥ 11.1mmol/L. Women who attended a minimum of one specialist appointment with an endocrinologist were categorised as ‘specialist diabetes care’ and all other women as ‘routine care’. Women were provided care as either ‘MNT’ (dietary management only) or ‘MNT + pharmacotherapy’ (insulin, as first-line therapy, or metformin, for women unable or declining to use insulin, when indicated by persistent hyperglycaemia; consistent with clinical practice guidelines at the Women’s Hospital).

### Data collection and variables

Sociodemographic and health outcome data was collected from medical records (parity, maternal age at delivery, infant sex and country of birth as a proxy for ethnicity). The Australian Bureau of Statistics’ (ABS) Standard Classification of Cultural and Ethnic Groups [19] was used to categorise country of birth. Socio-Economic Index for Areas (SEIFA) was derived from maternal postcode [20] with scores divided into quartiles (Quartile 1 = most disadvantaged, Quartile 4 = most advantaged). Early pregnancy body mass index (BMI) (kg/m²) measured at 16 weeks of gestation was classified according to World Health Organisation (WHO) criteria [21]. Health outcomes included ‘mode of delivery’, defined as either spontaneous/non-instrumental, instrumental birth (forceps and/or vacuum extraction), planned caesarean section or emergency caesarean section, ‘birth interventions’, defined as not induced (spontaneous and augmented labour), induced labour or no labour. ‘All maternal complications’ included instrumental birth, planned and emergency caesarean section and induced birth. ‘Neonatal complications’ included shoulder dystocia, stillbirth, respiratory distress and neonatal hypoglycaemia, admission to neonatal intensive care unit (NICU) or special care unit (SCN) and infant birth weight. Birthweight percentiles were calculated using Australian Birth Weight charts [22] and categorised into large for gestational age (LGA) and small for gestational age (SGA) when birthweight was > 90th centile and < 10th centile, respectively. Premature birth (delivery of live born infant < 37 weeks gestation) was determined from gestational age at delivery.

At The Women’s Hospital, English-speaking women with GDM are scheduled for an initial group education session, led by a dietitian, to obtain dietary advice about carbohydrate types and portions to consume, daily food group requirements and meal planning strategies. Individual review appointments with a dietitian are scheduled two weeks after the initial group education session for review of the food diary and blood glucose record. The dietitian scheduled a ‘second review’ appointment for women reporting uncertainty or worry about their dietary management or indicated over-restriction of dietary intake. Further appointments were made by the consulting dietitian on an individual basis, using the same criteria. Culturally and linguistically diverse women received interpreter-assisted dietetic consultations for the initial education session and at least one review appointment, with additional review appointments scheduled as needed, using the aforementioned criteria. The number of dietetic consultations attended for each woman was determined through patient management system audits and categorised into levels ‘no dietetic consultations, ‘one dietetic consultation’ ‘two dietetic consultations’ or ‘three or more dietetic consultations. Women with GDM were also taught to self-monitor their capillary blood glucose levels (BGLs), which were reviewed weekly by the Credentialed Diabetes Educator, Obstetrician or Endocrinologist (depending on model of care). Pharmacotherapy (insulin or metformin) was initiated when indicated by persistent hyperglycaemia consistent with clinical practice guidelines at the Women’s Hospital. It should be noted that complete data for maternal glycaemic control were not available for this study and could not be included in the analysis.

### Data analysis

Descriptive analyses were reported as mean(SD). Outliers were replaced with the next value that was not an outlier [23]. For categorical data, frequencies and percentages were reported. Logistic regression models were fitted for each binary maternal and neonatal outcomes, with the number of consultations as the independent variable. In one set of models, the number of dietetic consultations was included as a categorical variable; where there was evidence of an overall differences between groups (at the p < 0.05 level) then pairwise comparisons between number of dietetic consultation groups were tested. Moderation analyses were conducted whereby logistic regression models were fitted for each binary maternal and neonatal outcomes, with the number of consultations (categorical), pharmacotherapy (yes/no), and their interaction included as independent variables. Where there was evidence against the null hypothesis for the interaction factor (at *p* < 0.05), stratified analyses were conducted to assess effects of consultations for MNT and MNT + pharmacotherapy separately. Confounders were identified and adjusted for in the analyses, consistent with the literature [24–26]. For maternal complication outcomes, maternal age, early pregnancy BMI, gestational age at delivery, country of birth, LGA and previous caesarean section were adjusted for in the analyses. Maternal age, early pregnancy BMI, gestational age at delivery (except for the preterm birth outcome), country of birth, parity status (dichotomous), hypertensive disorders and infant sex were adjusted for infant outcomes [24, 26]. Data were analysed using Stata/SE 15 (StataCorp, TX).

## Results

### Participants

Of 1509 adult women with GDM, 1,185 were included in the analyses. Exclusions included prior dietetic intervention for other obstetric issues (n = 83), antenatal care (including GDM care) received outside The Women’s Hospital, Melbourne (n = 59), multiple pregnancy (n = 47), self-reported polycystic ovarian syndrome (n = 38), inpatient admission for serious medical conditions (n = 36), incomplete data (n = 23), no multidisciplinary care received (n = 20), diagnosis outside the ADIPS diagnostic criteria (n = 8), number of dietetic consultations could not be determined (n = 1). Where women had two pregnancies during the study period (n = 9), data for the first pregnancy were included.

Participant characteristics are shown in Table 1. Mean age of women was 32.4 years. Mean BMI was 27.4 kg/m². 19% of women received GDM care according to the U.S evidence-based clinical practice guidelines of a minimum of three dietetic consultations. Half of the sample received two dietetic consultations and 9% did not see a dietitian. For women who attended three or more dietetic consultations, almost twice as many were managed by MNT + pharmacotherapy (66%) compared with MNT alone (34%).

**Table 1:** Participant characteristics^a^

	Number of consultations				
	None (n = 101)	One (n = 275)	Two (n = 587)	Three or more (n = 222)	p-value
Maternal age at delivery (years)	31.8 ± 5.0	32.3 ± 5.2	32.6 ± 4.6	32.7 ± 4.4	0.34
Early pregnancy BMI (kg/m^2^)	28.8 ± 8.5	26.5 ± 6.2	26.3 ± 5.7	27.9 ± 7.1	**< 0.001** ^**b**^
Gestational age at delivery (weeks)	38.2 ± 1.3	38.4 ± 1.2	38.5 ± 1.1	38.1 ± 1.1	**< 0.001** ^**c**^
Infant sex					0.51
Female	50 (49.5%)	135 (49.1%)	277 (47.2%)	118 (53.2%)	
Male	51 (50.5%)	140 (50.9%)	310 (52.8%)	104 (46.8%)	
Parity status					**< 0.001**
0	36 (35.6%)ˇ	111 (40.4%)ˇ	319 (54.3%)ˆ	117 (52.7%)	
1	30 (29.7%)	92 (33.5%)	171 (29.1%)	60 (27.0%)	
2	14 (13.9%)	40 (14.5%)	54 (9.2%)ˇ	27 (12.2%)	
3	10 (9.9%)ˆ	14 (5.1%)	28 (4.8%)	10 (4.5%)	
4	11 (10.9%)ˆ	18 (6.5%)ˆ	15 (2.6%)ˇ	8 (3.6%)	
Model of care					**< 0.001**
Routine GDM care	83 (82.2%)	238 (86.5%)ˆ	506 (86.2%)ˆ	148 (66.7%)ˇ	
Specialist GDM care	18 (17.8%)	37 (13.5%)ˇ	81 (13.8%)ˇ	74 (33.3%)ˆ	
Type of therapy					**< 0.001**
MNT	59 (58.4%)	154 (56.0%)	314 (53.5%)	76 (34.2%)ˇ	
MNT + Pharmacotherapy	42 (41.6%)	121 (44.0%)	273 (46.5%)	146 (65.8%)ˆ	
Time of diagnosis					**< 0.001**
Routine diagnosis	100 (99.0%)ˆ	262 (95.3%)ˆ	556 (94.7%)ˆ	170 (76.6%)ˇ	
Early diagnosis	1 (1.0%)ˇ	13 (4.7%)ˇ	31 (5.3%)ˇ	52 (23.4%)ˆ	
	SEIFA Quartile				
	1 = most disadvantaged (n = 145)	2 (n = 451)	3 (n = 313)	4 = most advantaged (n = 276)	**0.049**
None	18 (12.4%)	38 (8.4%)	26 (8.3%)	19 (6.9%)	
One	34 (23.5%)	116 (25.7%)	79 (25.2%)	46 (16.7%)	
Two	68 (46.9%)	219 (48.6%)	156 (49.8%)	144 (52.2%)	
Three or more	25 (17.2%)	78 (17.3%)ˇ	52 (16.6%)	67 (24.3%)ˆ	
	Cultural Background				
	Australia and NZ (n = 333)	Europe and the Americas (n = 78)	African and Middle Eastern (n = 199)	Asian and other Oceania (n = 575)	**< 0.001**
None	37 (11.1%)ˆ	3 (3.9%)	26 (13.1%)ˆ	35 (6.1%)ˇ	
One	55 (16.5%)ˇ	10 (12.8%) ˇ	65 (32.7%)ˆ	145 (25.2%)	
Two	182 (54.7%)ˆ	47 (60.3%)	74 (37.2%)ˇ	284 (49.5%)	
Three or more	59 (17.7%)	18 (23.1%)	34 (17.1%)	111 (19.3%)	

^a^Descriptive statistics are shown as Mean ± SD for continuous measures and n (%) for binary or categorical measures; n = 1185

^b^Post-hoc tests indicated evidence (p < 0.05) of higher Early pregnancy BMI among those having zero or three or more consultations compared to those having one or two

^c^Post-hoc tests indicated evidence (p < 0.05) of higher gestational age among those having two consultations compared to those having zero, and lower gestational age among those having three or more consultations compared to those having one or two

ˆPost-hoc examination revealed higher than expected cell frequency (adjusted residual for cell > 1.96; p < 0.05)

ˇPost-hoc examination revealed lower than expected cell frequency (adjusted residual for cell < -1.96; p < 0.05)

### Dietetic consultations and maternal complications

Associations between dietitian consultations and maternal complications are presented in Table 2. Most women (81%) experienced one or more maternal complication. Some evidence of group differences for the ‘all maternal complications’ outcome was seen (p = 0.04). Pairwise comparisons showed significant higher odds of (any) maternal complication among women receiving 3 + consultations compared with those receiving zero (OR = 2.33 [95% CI: 1.23, 4.41], p = 0.009), one (OR = 1.80 [95% CI: 1.09, 2.98], p = 0.02), or two (OR = 1.65 [95% CI: 1.04, 2.60], p = 0.03) consultations.

**Table 2:** Association between level of dietetic intervention and maternal complications^a^

Maternal outcomes	Level of dietetic intervention (no. consultations)				p-value for overall group differences	p-value for interaction of pharmacotherapy (yes/no) and level of dietetic intervention
	None	One	Two	Three or more		
All maternal complications	77 (76.2%)	216 (78.5%)	472 (80.4%)	193 (86.9%)	0.04	0.41
Unplanned caesarean section	18 (17.8%)	39 (14.2%)	75 (12.8%)	38 (17.1%)	0.54	0.99
Planned caesarean section	25 (24.8%)	54 (19.6%)	115 (19.6%)	49 (22.1%)	0.43	0.60
Induced labour	44 (43.6%)	130 (47.3%)	309 (52.6%)	122 (55.0%)	0.30	0.17
Instrumental birth	11 (10.9%)	38 (13.8%)	112 (19.1%)	37 (16.7%)	0.49	0.47

^a^All models were adjusted for maternal age, early pregnancy BMI, gestational age at delivery, cultural background, whether baby was large for gestational age, and whether mother had a previous caesarean section; n = 1185

### Dietetic consultations and neonatal complications

Overall, 18% of women had one or more neonatal complication (Table 3). Some evidence of group differences for the admission to NICU/SCN outcome was seen (p = 0.02). Pairwise comparisons showed significantly lower odds of admission to NICU/SCN among babies of women receiving one (OR = 0.38 [95% CI: 0.18, 0.78], p = 0.008), two (OR = 0.37 [95% CI: 0.19, 0.71], p = 0.003), or 3 + consultations (OR = 0.43 [95% CI: 0.21, 173 0.88], p = 0.02) compared with those receiving zero. There was also some evidence of group differences for the premature birth outcome (p = 0.02). Pairwise comparisons showed significantly lower odds of premature birth among babies of women receiving two consultations compared with those receiving zero (OR = 0.35 [95% 179 CI: 0.15, 0.78], p = 0.01), one (OR = 0.49 [95% CI: 0.25, 0.95], p = 0.04), or 3 + consultations (OR = 0.44 180 [95% CI: 0.22, 0.86], p = 0.03).

**Table 3:** Association between level of dietetic intervention and neonatal complications^a^

Neonatal outcomes	Level of dietetic intervention (no. consultations)				p-value for overall group differences	p-value for interaction of pharmacotherapy (yes/no) and level of dietetic intervention
	None	One	Two	Three or more		
All neonatal complications	28 (27.7%)	46 (16.7%)	90 (15.3%)	48 (21.6%)	0.28	0.65
Admission to NICU/SCN	22 (21.8%)	25 (9.1%)	43 (7.3%)	28 (12.6%)	0.02	0.51
Neonatal hypoglycaemia	6 (5.9%)	16 (5.8%)	36 (6.1%)	13 (5.9%)	0.99	0.86
Shoulder dystocia	3 (3.0%)	1 (0.4%)	10 (1.7%)	4 (1.8%)	^b^	^b^
Respiratory distress	2 (2.0%)	8 (2.9%)	3 (0.5%)	4 (1.8%)	^b^	^b^
Large for gestational age	16 (15.8%)	22 (8.0%)	51 (8.7%)	21 (9.5%)	0.66	0.53
Small for gestational age	9 (8.9%)	24 (8.7%)	34 (5.8%)	22 (9.9%)	0.15	0.42
Premature birth (< 37 weeks gestation)	10 (9.9%)	18 (6.5%)	20 (3.4%)	16 (7.2%)	0.02	0.23

^a^ All models were adjusted for maternal age, early pregnancy BMI, cultural background, parity status (dichotomous), hypertensive disorders, and the infant’s sex. Gestational age at delivery was adjusted for in models for all outcomes apart from premature birth; n = 1185

^b^ Inferential analyses were not conducted for these outcomes due to the low occurrences rate in neonates

## Discussion

Overall, 19% of women in this study received at least three dietetic consultations during pregnancy and of these women, almost twice as many were managed by MNT + pharmacotherapy (66%) compared with MNT alone (34%). A high proportion of women in this study experienced maternal complications. Further, compared to women who did not see a dietitian during pregnancy, for those who did see a dietitian, lower odds of admission to NICU/SCN were observed. Whilst half of the women in our study saw a dietitian twice during pregnancy, less than one fifth of women in our study received GDM care according to the U.S evidence-based clinical practice guidelines of a minimum of three dietetic consultations. However, no evidence was shown that women who were seen in accordance with these recommendations had better maternal and neonatal health outcomes. This suggests that the current recommendations regarding schedule of consultations requires re-examination.

The U.S MNT guidelines were developed for a contrasting health system and importantly, the schedule for the number of consultations was derived from expert consensus only [15]. The guidelines recognise several barriers that may inhibit access to healthcare services including financial burdens, inability to take time off work, and lack of childcare or transportation but they appear to be unrealistic for current Australian practice [15]. There is a need to explore alternative and innovative models of care for women with GDM. For example, implementing different levels of care for women who are triaged according to complexity of their GDM may be an efficient, alternative model for management of these women. Wong and colleagues [27] explored this approach by stratifying women with GDM into two treatments groups; intensive (n = 46) or conservative (n = 35) management, based on diagnostic OGTT results. There was no difference in mean birthweight, rates of macrosomia or LGA between groups [27]. This suggests that stratification of women at diagnosis of GDM, might assist effective and safe management whilst utilising less healthcare resources.

The mode of dietetic care delivery for MNT is another area that should be re-evaluated. The U.S MNT practice guidelines recommend that consultations be individual rather than group-based [28]. Yet currently only about a third of Australian GDM cases are seen individually, due to excessively high demand [29]. While not aligned with the MNT guidelines, previous research has found group education to be effective in increasing nutrition knowledge and more time effective for dietitians [30, 31]. Furthermore, telehealth management has been shown to result in improved glycaemic control [32–34], higher levels of blood glucose testing, and lower rates of pharmacotherapy [32]. The COVID-19 pandemic has forced health services to pivot consultations into digital delivery modes and significant progress has been made regarding clinical efficiencies and versatility in delivering information to vulnerable pregnant populations [35]. While insufficient evidence exists to determine if telehealth is more or less effective in improving maternal and neonatal outcomes [36], web-based and digital methods offer potential for enhanced service delivery and wide-reach across diverse populations groups. The views of pregnant women regarding telehealth services are not fully understood, yet limited data so far shows that women find it to be a positive experience overall [35, 37]. While telehealth may assist workload flexibility, [27, 38] further research is needed to determine its impact on pregnancy outcomes across population groups.

The need to ‘modify’ and ‘modernise’ approaches to GDM care for women has emerged as a critical priority in Australian healthcare [4]. Whilst inadequate funding to support greater practitioner utility and higher levels of staffing has been acknowledged as a major roadblock to provision of care for women with GDM [4], there is an urgent need to invest in relieving healthcare systems which are failing to adequately meet the needs of a large proportion of women in Australia. Of women in our study who received 3 + dietetic consultations, almost twice as many were managed by MNT + pharmacotherapy, compared with MNT alone. This indicates that women who require greater dietetic input were experiencing difficulty in managing their glycaemia and overall, required more resource intensive management. Irrespective of whether women were managed by pharmacotherapy, the number of consultations with a dietitian was not significantly associated with risk of maternal complications. In fact, most women in our study (81%) experienced one or more maternal complications and those who did, were more likely to receive 3 + consultations. The impact of dietetic input on neonatal outcomes is less clear. Women who saw a dietitian at least once during pregnancy had babies with a lower risk of NICU admission, and might be attributed to the underlying patient risk characteristics and overall GDM clinical management required for women who had three or more consultations, rather than a lack of dietetic impact.

### Strengths and limitations

There are both strengths and limitations of this study. Firstly, we included a large and culturally diverse sample of women with GDM who gave birth at a large metropolitan maternity hospital in Melbourne. We adjusted for multiple, important confounders in our analyses which strengthened the observed relationship between number of dietetic consultations and maternal and neonatal outcomes. However, we cannot infer causality from our results due to the retrospective study design. We were not able to obtain glycaemic management data which is an important limitation as this and would likely have influenced both maternal and neonatal outcomes. Further, as this was a retrospective study we were unable to assess dietary intake change following group education or individual dietetic management. Dietary change is a key outcome and should be considered in future research alongside assessment of maternal and neonatal health outcomes in women with GDM.

## Conclusion

The optimum number of consultations to achieve best maternal and neonatal health outcomes remains uncertain. An individualised approach to GDM management is necessary to achieve optimal pregnancy outcomes. Current MNT guidelines lack applicability to differing health resource settings such as Australia. Future research into alternate methods for the delivery of MNT are needed to ensure health services have the capacity to deliver evidence-based care to women with GDM.

## Data Availability

The datasets used and/ or analysed during the current study are available from the corresponding author on reasonable request.

## Abbreviations

GDM: Gestational Diabetes Mellitus
BMI: Body Mass Index
MNT: Medical Nutrition Therapy
NICU: Neonatal Intensive Care Unit
